# The Likeness between Alcoholism and General Paralysis of the Insane

**Published:** 1899-09-09

**Authors:** 


### THE LIKENESS BETWEEN ALCOHOLISM AND GENERAL PARALYSIS OF THE INSANE.

In a lecture delivered at Bethlem Royal Hospital Dr. Savage[1] points out how allied to the symptoms of general paralysis of the insane are many of those produced by alcohol. First of all, however, it has to be borne in mind that there is a great deal of unsoundness of mind that cannot be treated as insanity, and yet the person affected is temporarily beside himself, and among these conditions are many which are due to alcoholism, among which may be placed delirium tremens and acute delirious mania produced by alcohol. Turning now to the similarity between the symptoms of alcoholism and of general paralysis of the insane: The same parts are affected, and in the same order. Alcohol affects the higher intellectual centres, and thus lead to defective control, and so also with general paralysis; while again the earliest symptoms of the latter condition may be hysterical, emotional, and sentimental, just as may be the case in alcoholic insanity. Then, as the result both of alcohol and of progressive brain defect in general paralysis of the insane, there is loss not alone of the highest mental control but of physical control, as shown by tremor. In alcoholism, indeed, one may meet with tremor of the tongue and lips, and of the hands, and also a hesitating gait, and these things may be even more clearly marked than in general paralysis. It is to be noted, however, as a distinction, that one rarely meets with tremors so well marked early in the general paralysis as one does in alcoholism. As a mental symptom also there is general instability, "to one thing constant never," which is common to both conditions. As to grandiose ideas, "occasionally the alcoholic is benevolent, giving away what does not belong to him, but as a rule he is not so benevolent as the general paralytic." Defective memory for recent events is also common to both affections. But with all this it is to be remembered that, although in the alcoholic dissolution may appear more marked than in the general paralytic, the alcoholic may get well. In reference to this, Dr. Savage said: "One of my favourite maxims is, 'To the alcoholic all things are possible.' I scarcely ever condemn a person, however bad he or she may be physically, as incurable, if there be a distinct history of alcoholism. I always think of being called down into Surrey to see a man, and when I looked at him lying on his back breathing stertorously, perfectly unconscious, and when further examination showed that he had bed-sores, ulceration of one heel, I thought him sinking, and I said to the general practitioner, 'Well, could they not let him die without my permission?' He said, 'No, the friends thought they would like you to see him before he went.' He told me the history, and I said, 'Well, of course, it looks to me as if he is going to die, but to the alcoholic all things are possible.' Four months afterwards a man tottered into my consulting-room between two sticks. He said, 'You do not remember me?' I said, 'No.' He said, 'To the alcoholic all things are possible.' I said, 'You did not remember that?' He said, 'No; but I was told when you were called in to see me die that you said, "To the alcoholic all things are possible."' He said, 'Do you think it is possible for me to return as sporting editor to a certain sporting paper?' I said, 'Well, it is possible, but not advisable.' I never heard of that gentleman since, but it is a very good example of what I refer to."

Among other products of alcoholism may be delusions, which, however, may have their origin in the perverted sensations which are produced by the peripheral neuritis which so commonly results from the consumption of alcohol, and these delusions may be associated with grand ideas in such a way as still further to increase the similarity between alcoholism and general paralysis. In attempting to differentiate these conditions, first there is the question of sleep. The general paralytic generally sleeps better than the alcoholic. Then the paralytic has generally some inequality of pupils, with slowness of reaction. In alcoholism the tremor, which is a comparatively early symptom, passes off under abstinence; while, with the paralytic, both the tremor and the hesitancy of speech tend to increase with time. Then there is the question of the reflexes. In general paralytics there is generally a very marked alteration either in the direction of excess or defect. This criterion, however, may be confused by the occurrence of peripheral neuritis in alcoholics, when the reflexes also may be altered. Finally, a condition of exaltation may be met with in both, but the exaltation of the general paralytic is more universal and more benevolent than in that of the alcoholic. Two further points in regard to the relation between alcoholism and general paralysis are worth bearing in mind. First, that general paralysis is not very commonly produced by drink alone, and next that symptoms obviously the immediate results of alcohol may be really indicative of general paralysis. Thus, the general paralytic is very readily affected by alcohol. "I have," says Dr. Savage, "known a man who had lived freely, and had been able to take any amount of alcoholic stimulants, nevertheless begin to show the symptoms of the general paralytic after taking a glass of wine. He was induced to get up and return thanks for the health of the Queen, and the single glass of wine had made this man, who had yet been a hard drinker, drunk. This instability is one of the symptoms of the general paralytic."

Dr. Savage wisely says, "It is a very valuable axiom that no person suffering from mental disorder is well till he recognises that he has been ill, and is also grateful for the treatment. One of the proofs that nearly all alcoholics are only relieved and not cured is that they are scarcely ever cognisant of their illness, or grateful for what has been done for them."

This has some bearing upon the question of certifying, for a man may be brought into hospital suffering from acute alcoholism, or insanity depending upon acute alcoholism, and in a few days the whole symptoms may pass off. "If I am asked," says Dr. Savage, "to see an alcoholic patient, however violent and however threatening . . . I hesitate to certify to his insanity." He gives a case, and adds, "It is all very well, but I am not going to sign a certificate for a patient suffering only from acute alcoholism. What would be the result? You send a patient into an asylum. In forty-eight hours he is better; at the end of a week he is so far well as to recognise where he is and what you have done. The only people who have ever prosecuted me legally for having a hand in their detention in Bethlem, or in their removal to asylums, have been people suffering in that way," which is a thing to remember, for sometimes one is sorely tempted to shut such a patient up.

[1] Clinical Journal, Aug. 30.

